# Haze-protective self-care behaviours in Sarawak, Malaysia: a state-representative cross-sectional study

**DOI:** 10.1038/s41598-026-49870-9

**Published:** 2026-04-20

**Authors:** Sam Froze Jiee, Lim Jyh Hann, Lim Siong Hee, Romano Ngui, Timothy Adrian Joseph, Neilson Richard Seling, Anselm Su Ting, Jeffery Stephen

**Affiliations:** 1 Department of Community Medicine and Public Health, Faculty of Medicine and Health Sciences, Universiti Malaysia Sarawak (UNIMAS), Kota Samarahan, Sarawak Malaysia; 2 Environmental Health Sector, Disease Control Division, Ministry of Health Malaysia, Putrajaya, Malaysia; 3 Department of Paraclinical Sciences, Faculty of Medicine and Health Sciences, Universiti Malaysia Sarawak (UNIMAS), Kota Samarahan, Sarawak Malaysia

**Keywords:** Transboundary haze, Protective self-care behaviours, Community resilience, Ordinal logistic regression, Sarawak, Borneo, Diseases, Environmental social sciences, Health care, Risk factors

## Abstract

Transboundary haze is a recurrent hazard in Borneo that elevates particulate exposure to health-relevant levels. We conducted a community-based cross-sectional survey of adults across Sarawak, Malaysia, using multistage sampling (n = 481) to assess haze-related protective self-care behaviours during haze. The primary outcome was a Likert-based self-care index categorised into Poor/Moderate/Good using sample quartiles. Determinants included haze tolerance score (as operationalised in this study), political trust, perceived costs, haze knowledge, and community resilience, alongside sociodemographic and access indicators. Analyses included zonal comparisons and proportional odds regression with sensitivity analyses using multinomial regression and the continuous self-care score. Only 25.6% achieved good self-care, while 49.3% were moderate; self-care differed by zone, with the Northern zone scoring highest despite lower trust/knowledge profiles. In adjusted ordinal models, proximity to a health facility was associated with a higher self-care category (OR 3.38, 95% CI 2.17–5.27; *p* < 0.001), while unmarried status was associated with a lower category (OR 0.48, 95% CI 0.28–0.82; *p* = 0.007). Higher tolerance score (OR 1.18, 95% CI 1.09–1.28; *p* < 0.001) and haze knowledge (OR 1.18, 95% CI 1.07–1.30; *p* = 0.001) were positively associated, whereas community resilience showed a small inverse association (OR 0.96, 95% CI 0.93–0.99; *p* = 0.013). These findings underscore the importance of actionable know-how and practical access to support higher self-care categories. A trigger-based playbook linking API/PM2.5 thresholds to behaviourally specific messaging and provision of effective respirators and clean-air options warrants evaluation to strengthen population protection during seasonal haze.

**Supplementary Information:**

The online version contains supplementary material available at 10.1038/s41598-026-49870-9.

## Introduction

Landscape fire smoke is a persistent global public health hazard. Contemporary reviews synthesise the multi-system health effects of wildfire-related Particulate Matter (PM) 2.5 and document the rising population exposure driven by climatic variability and land-use change, indicating that smoke episodes will remain recurrent despite mitigation efforts^1,2^. In this context, “landscape fire smoke” refers to smoke from the open burning of vegetation and peatlands (including local and transboundary fires), which produces fine particulate matter and other combustion-related pollutants that can persist and travel over large geographic areas^2^. In Asia, long-lived plumes from tropical biomass burning, particularly those from drained peatlands, drive seasonal air quality extremes. Recent atmospheric syntheses emphasize sustained peat sources and plume chemistry that enable long-range transport and secondary aerosol formation^3^.

Within Southeast Asia, seasonal transboundary haze remains a defining environmental-health challenge, with consistent evidence of acute cardiorespiratory morbidity and mortality during severe episodes^1,4^. In recognition that haze risk persists, ASEAN adopted the Second Haze-Free Roadmap (2023—2030) to strengthen prevention and explicitly elevate public health protection, underscoring the need for preparedness alongside suppression^5,6^. Sarawak provides a particularly policy-relevant setting for haze preparedness because its communities span dense urban centres, dispersed rural settlements, and remote longhouse areas with substantial outdoor livelihoods and variable access to health services and protective resources. These contextual differences may shape both exposure-reduction feasibility (for example, the ability to remain indoors and access clean-air spaces) and the uptake of recommended self-care actions. We therefore examined patterns across Sarawak’s zones to identify inequities and inform more targeted, operationally feasible risk-reduction strategies.

On Borneo Island, recurrent peat-fire activity in Kalimantan can transport smoke across borders, contributing to region-wide air quality deterioration during severe haze seasons, such as in 2019^4,7^. In Sarawak, monitoring station reports documented repeated days of unhealthy air quality during the 2023 haze season (September–October), and these episodes coincided with satellite-observed smoke movement from Kalimantan. This aligns with published analyses reporting pronounced episodic haze signals in regional particulate matter during transboundary smoke events^8–11^. Although more urbanised states such as Selangor, Kuala Lumpur, and Pulau Pinang often report persistently poorer air quality, largely driven by urban and industrial emissions, our study specifically examines protective behaviours during *seasonal haze* events dominated by landscape-fire smoke. Sarawak provides a distinct and policy-relevant haze context because episodic smoke intrusions can affect wide geographic areas, including dispersed rural and remote communities, where the feasibility of recommended self-care (staying indoors, accessing protective equipment, or reaching services) may differ from densely urban settings. In this sense, Sarawak offers an important setting for understanding behavioural determinants under real-world constraints and for informing preparedness strategies that remain applicable across heterogeneous settlement patterns.

The consequences of seasonal haze span health, well-being, the economy, and the health-care system. Short-term smoke exposure is associated with asthma and COPD exacerbations, increased hospital admissions, and health-care utilisation in Malaysia and the wider region; multi-site and time-series studies demonstrate higher demand during haze days^12,13^. Ocular effects are also documented, with population-level data from Singapore showing that haze exacerbates conjunctivitis associated with short-term particulate matter increases^14^. Beyond clinical outcomes, economic assessments attribute multi-billion-dollar health-related losses to major Southeast Asian fire-smoke crises, underscoring the scale of impact that extends beyond clinical endpoints^4^.

**Fig. 1:**
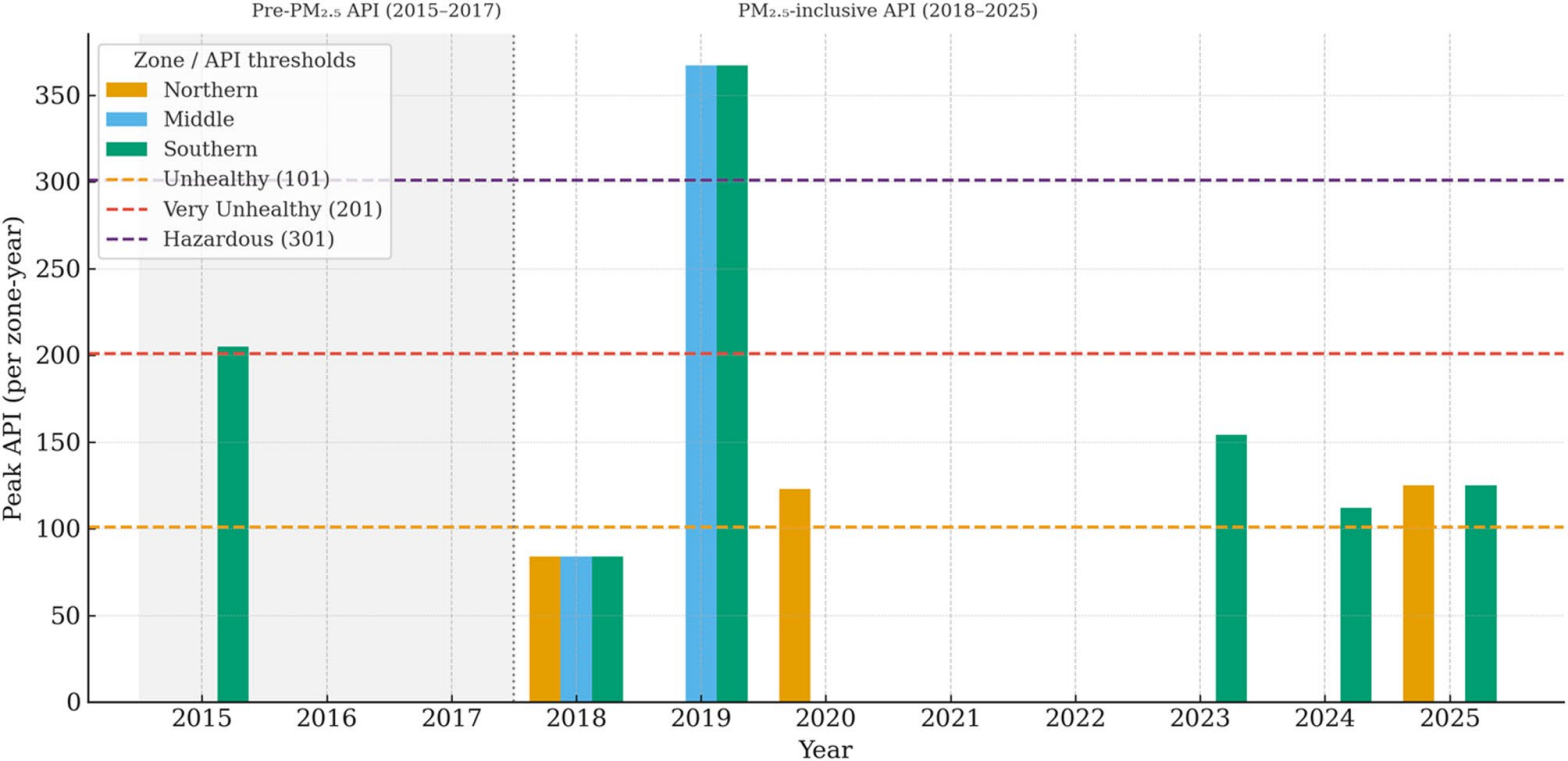
Peak annual Air Pollutant Index (API) by zone in Sarawak (2015–2025). Peak API values are shown for Southern, Central, and Northern Sarawak.

Transboundary haze is a persistent and recurring issue recognized internationally as a regional and global concern rather than just a temporary local nuisance^5,6^. In Malaysia, haze is not a new problem; historical analyses and official records show significant national episodes in 1997, 2005, 2013 and 2015, often exacerbated by El Niño conditions^15,16^. As shown in Fig. 1, peak annual API values differed across Sarawak’s three zones (Northern, Central, Southern) from 2015 to 2025, with several years where one or more zones exceeded the “Unhealthy” threshold and, in some years (2019), approached or exceeded “Very Unhealthy” levels. For context, Malaysian API categories commonly used for public health communication include Unhealthy (≥ 101), Very Unhealthy (≥ 201), and Hazardous (≥ 301)^17^. The figure also notes the 2018 change to a PM 2.5-inclusive API, indicating that values before 2018 are not directly comparable to those after; however, they reveal that all zones have experienced episodes of health-perturbing haze. Government responses involve multiple ministries and agencies, including the DOE (monitoring and advisories), the Ministry of Health (workplace and public health guidance), and national risk communication led by disaster management authorities; however, the success of communication and community engagement varies. Recent reviews and policy analyses emphasize the need to improve community-centred risk communication and resilience to promote protective behaviours during haze episodes^18–22^.

Situated in Sarawak, Malaysia’s largest state on the island of Borneo, the region is characterized by its diverse geography and varied livelihoods, including a significant proportion of outdoor employment. Nonetheless, the persistent seasonal haze remains a substantial public health challenge. For interpretability, Sarawak was analysed in three zones (Southern, Central, and Northern) to reflect meaningful geographic and sociodemographic heterogeneity. Southern Sarawak is generally more urbanised and densely settled. In contrast, Central and Northern Sarawak include larger rural and interior areas characterised by more dispersed settlements, longer travel distances, and greater reliance on outdoor livelihoods. These differences have practical implications for haze response, as the feasibility of recommended protective measures may vary by zone. Socioeconomic differences and varied settlement patterns lead to unequal exposure and capacity to respond, resulting in diverse adoption of recommended self-care practices. Because transboundary haze is a recurring, largely uncontrollable seasonal phenomenon, communities inevitably face periods of poor air quality while suppression and regulatory measures are implemented. This study aimed to measure the extent and patterns of protective self-care behaviours during haze episodes among adults in Sarawak and to identify the factors that influence these behaviours. Using locally grounded preliminary evidence on modifiable factors that drive protective actions across Sarawak’s diverse settings, the study seeks to guide targeted public health messaging and promote equitable, population-level risk-reduction strategies. Conceptually, we assumed that a sequence of determinants shapes protective self-care during haze: (i) risk appraisal and acceptance (haze tolerance), (ii) enabling cognition and skills (knowledge), (iii) institutional trust that influences message uptake (political trust), (iv) feasibility constraints (perceived costs and access to resources/services), and (v) community-level adaptive capacity (community resilience). These domains were measured to test whether modifiable psychosocial factors and structural access indicators are associated with higher self-care categories during haze.

## Methods

### Study design and setting

We undertook a community-based cross-sectional study across the state of Sarawak, Malaysia, to characterise population health and socio-economic indicators in relation to seasonal haze. The design prioritised statewide coverage and external validity by sampling from Sarawak’s major geographic belts, southern, central, and northern, which differ in settlement patterns, livelihoods (including outdoor work), and access to services.

### Eligibility criteria of study participants

The inclusion criteria comprised adults aged 18 years or older who currently lived in Sarawak, had resided continuously in the state for at least 12 months, and were able to provide informed consent. Exclusion criteria were (i) cognitive impairment precluding informed participation, (ii) an active mental-health condition that, in the judgment of the field team, would impede a safe or reliable interview, or (iii) critical illness at approach. To reduce intra-household correlations and respondent burden, we interviewed one eligible adult per household. When multiple eligible adults were present, the respondent was chosen using a standardised within-household selection rule to minimise selection bias and ensure consistent selection across households. These criteria were chosen to ensure that participants had established exposure to local haze conditions and community context, could understand and voluntarily consent to study procedures, and could complete interviewer-assisted instruments without undue risk. Language support was provided through trained multilingual Field Research Assistants; individuals unable to communicate adequately, even with assistance, were not enrolled. Substitutions were not permitted once eligibility and consent were determined, and ineligible/declined cases were logged for recruitment accounting.

### Sample size calculation and sampling method

The minimum required sample size was 462, computed for a Sarawak population of approximately 2.4 million^23^, with a 5% margin of error, a 95% confidence level, and a 50% response distribution to maximise variability. This figure was then inflated by 20% to account for non-response/attrition. The calculation followed the standard survey formula as implemented in the Raosoft Sample Size Calculator^24^. The finite-population correction is negligible at this population size; nonetheless, the achieved sample was planned to meet or exceed the minimum to ensure precision within regional strata.

**Fig. 2:**
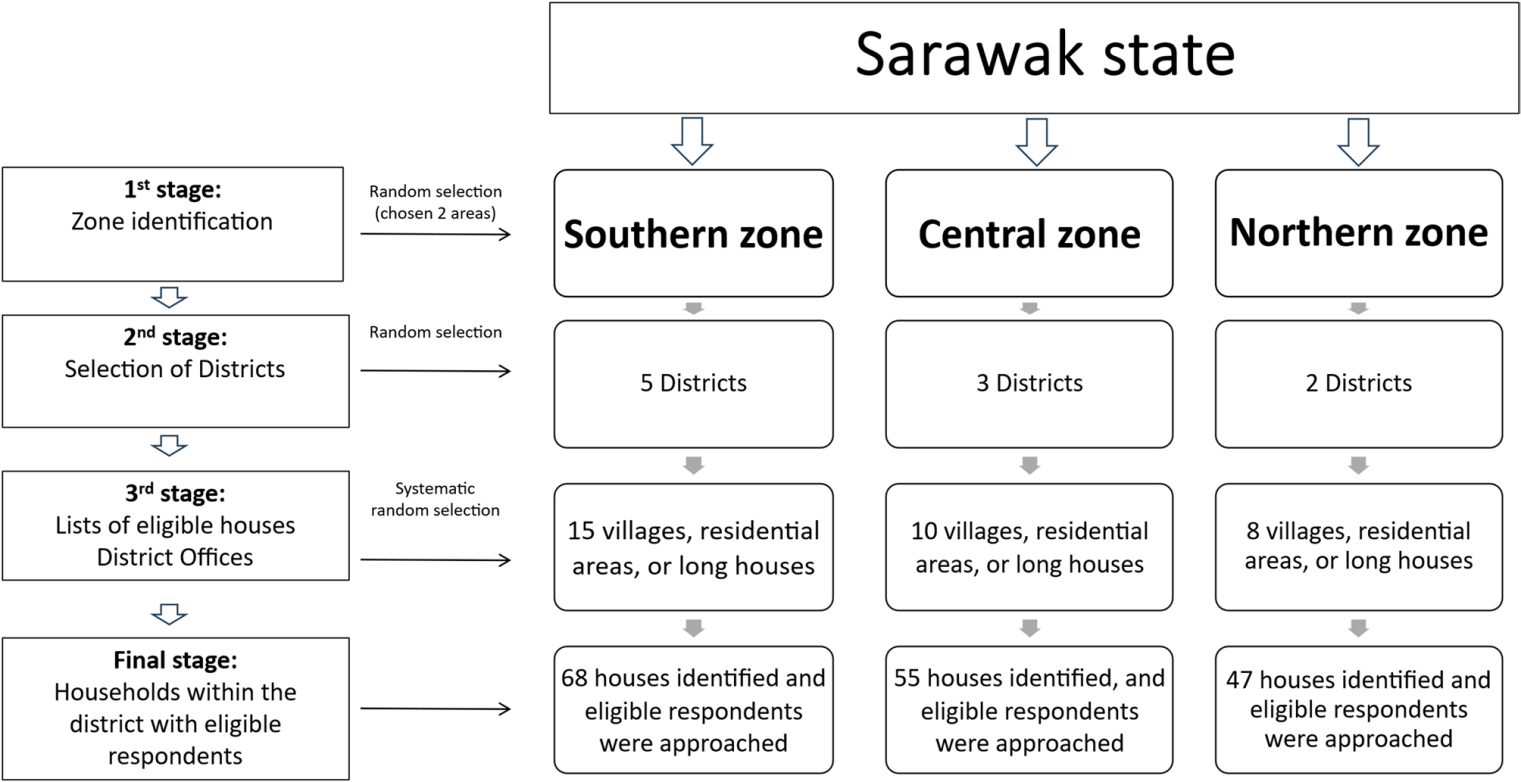
Multistage stratified cluster sampling across Sarawak’s Southern, Central, and Northern zones, with random district and community-cluster selection followed by systematic household sampling.

We utilised a multistage, stratified cluster sampling design aligned to the flow shown in Fig. 2. Stage 1 (Zone Identification): Sarawak is administratively divided into three zones: Southern, Central, and Northern. This convention is consistently used across state planning and sectoral documents, ensuring geographic and socio-demographic representation^25^. Stage 2 (district selection): Within zones, districts were randomly selected, yielding five in the southern zone, three in the central zone, and two in the northern zone. Stage 3 (cluster selection): for each selected district, we obtained official lists of eligible households from the District Offices and sampled community clusters (villages, residential areas, or longhouses) as follows: 15 clusters in the southern zone, 10 in the central zone, and 8 in the northern zone. From each district list, a systematic random sample of 52 households was then selected (using a fixed sampling interval with a random start). Stage 4 (households/respondents): field teams visited sampled households and interviewed at least one eligible adult; when multiple eligible adults were present, one respondent was selected using a pre-specified within-household rule.

### Questionnaire development, validation, scoring, and administration

The instrument comprised five sections: a combination of newly developed items and items adopted from previously published studies and guidance relevant to haze risk communication and protective practices. Where adaptations were made, item wording was refined to improve contextual relevance to Sarawak while preserving the intended construct. The instrument comprised five analytic sections: Part 1 (Sociodemographic characteristics) captured age, sex, marital status, ethnicity, religious practices, education, household size, living arrangements, housing/residence type, and locality (urban, suburban/semi-urban, rural); Part 2 (Medical and mental-health history) recorded self-reported diagnoses and symptoms pertinent to haze susceptibility, particularly respiratory conditions, with space for open-ended specification of other ongoing issues. Haze tolerance in this study referred to the degree to which individuals accept or normalise haze episodes and perceive less need to undertake protective actions; Part 3 (Haze-related determinants) assessed constructs theorised to influence protective action, haze tolerance (acceptability of haze, willingness to pursue outdoor activities without a mask or with exercise, absence of indoor purification), political trust (perceived control capacity, transparency/appropriateness of official information and measures, confidence in future innovations), perceived costs (economic and mobility/traffic), and knowledge (information-seeking intention and perceived understanding of causes, health effects, and recommended controls), all rated on 5-point Likert scales (strongly disagree to strongly agree); Part 4 community resilience was assessed as a scale-based construct capturing perceived community connection/caring, available resources, transformative potential, and disaster-management capacity using 5-point Likert items^26^; and Part 5 (Self-care behaviours related to haze), the primary outcome, organised under Health Belief Model domains (perceived severity, susceptibility, benefits, barriers, self-efficacy, cues to action) alongside specific practices (staying indoors during emergency/peak haze, using face masks/respirators) and aligned with recent HBM-based self-care instruments for air-pollution protection^20^. For Part 5 (primary outcome: self-care behaviours related to haze), we calculated each participant’s per-item average across all behaviour items (1–5 scale, reverse-coded).

We used sample-derived quartiles to categorise the composite self-care score because there are no established guideline-based cut-offs for this multi-behaviour composite and because quartiles enable clear within-sample stratification for programme planning. We acknowledge that quartile thresholds are data-dependent and therefore interpret Poor/Moderate/Good categories as relative groupings within this population rather than externally generalisable cut-offs. The categorical outcome was then derived using the sample’s empirical quartiles of this Part-5 average: Poor (≤ 25th percentile), Moderate (25th to < 75th percentile), and Good (≥ 75th percentile); tied values at cut-points were assigned to the more conservative (lower) category. We categorized the primary outcome to provide policy-relevant, easily interpretable bands that accommodate non-linear associations and skewed distributions, enabling clear risk stratification and comparison across subgroups^27^. For interpretability, we coded the haze tolerance domain so that higher scores indicate lower tolerance for risky haze exposure (a greater inclination to avoid exposure and adopt precautionary actions), rather than acceptance of haze conditions. Internal consistency of multi-item scales was assessed using Cronbach’s alpha; values for each domain (haze tolerance, political trust, perceived costs, knowledge, community resilience subdomains, and the Part-5 self-care index).

Following translation of the validated instrument into Malay, the questionnaire underwent content validation by experts from the Department of Community Medicine and Public Health, Faculty of Medicine and Health Sciences, Universiti Malaysia Sarawak, and Public Health Medicine Specialists from the Ministry of Health Malaysia, to ensure relevance, clarity, and cultural appropriateness. A pilot study (n = 45) was then conducted to assess internal consistency reliability using Cronbach’s alpha. Pilot testing was carried out in Lundu District and Padawan Subdistrict, selected because these localities include diverse ethnic and socio-economic groups that reasonably replicate the heterogeneity of the actual study population and field conditions. Overall, the domains demonstrated acceptable to excellent internal consistency: haze tolerance (4 items; α = 0.718) and protective self-care behaviours during hazes (25 items; α = 0.726) showed acceptable reliability; cost perception (3 items; α = 0.793) showed good reliability; and political trust (4 items; α = 0.920), community resilience (14 items; α = 0.918), and haze knowledge (5 items; α = 0.952) showed excellent reliability. While very high alpha values (for instance, haze knowledge) may reflect highly overlapping item content, we retained all items to preserve theoretical coverage and content validity, particularly given the modest pilot sample size.

Data were collected through face-to-face, interviewer-assisted household interviews conducted by the research team (Researchers and Field Research Assistants) who were fluent in Sarawakian Malay dialect, Iban, and Mandarin, allowing respondents to participate in their preferred language. Formal inter-rater reliability testing was not performed. However, we ensured interviewer standardization by implementing structured training using standardized scripts and prompts, along with field supervision that included routine reviews of completed questionnaires. This approach aimed to maintain consistent interview administration across different interviewers and languages. Interviewers adhered to a standardized field manual and used scripted prompts, ensuring that written informed consent was obtained before each interview. Interviews were conducted in a private area of the respondents’ homes to protect confidentiality. To maintain consistency and minimize interviewer bias, all staff members underwent centralized training focused on neutral probing, respondent safety, cultural sensitivity, and distress management. Competency was verified through supervised practice sessions. Interviewers read each question verbatim, recorded responses contemporaneously on encrypted tablets and paper, and implemented real-time logic and range checks; supervisors performed same-day reviews and random back-checks by phone or revisit to verify key items and consent. Up to two return visits at different times of day were attempted for non-contacts; refusals were not replaced.

To minimise selection bias, replacements were permitted only for closed or vacant dwellings after at least two return visits at different times; refusals were not replaced. Community liaison assisted with wayfinding and listing verification in sparsely populated areas, and daily logs recorded non-contacts, ineligibles, and refusals. This design achieved statewide coverage across urban, suburban/semi-urban, and rural/remote areas while accounting for intracluster correlation. Analyses, therefore, included prespecified robust variance estimation and design-effect sensitivity checks.

### Statistical analyses

We summarised categorical variables as frequencies (%) and continuous variables as mean (SD) or median (IQR), as appropriate; zonal comparisons used chi-square tests for categorical variables and ANOVA for continuous variables. The primary outcome (Poor/Moderate/Good self-care category) was modelled using proportional-odds ordinal logistic regression to estimate the cumulative odds of being in a higher self-care category. The proportional-odds (parallel-lines) assumption was assessed using the Test of Parallel Lines, which indicated a violation (LR χ^2^ (5) = 88.33, *p* < 0.001); therefore, we conducted multinomial (generalised logit) regression as a sensitivity analysis and interpreted proportional-odds estimates as summary associations supported by threshold-specific results. Sensitivity analyses treating the self-care score as a continuous outcome yielded consistent directions and significance for key predictors, indicating that conclusions were not driven by categorisation. Multicollinearity was assessed using tolerance and variance inflation factors (VIF) and was not a concern (tolerance 0.379–0.695; VIF 1.439–2.635). Analyses were performed using SPSS version 29.0 with two-sided significance at *p* < 0.05.

### Ethical considerations

The study complied with institutional and national research ethics requirements and with the principles outlined in the Declaration of Helsinki. Ethical approval was granted by the Medical Research Ethics Committee of Universiti Malaysia Sarawak (UNIMAS) (Reference: FME/24/129, 7 May 2024). Participation was entirely voluntary, and written informed consent was obtained before data collection commenced. Before obtaining consent, interviewers explained the study’s purpose, procedures, expected duration, potential risks/benefits, data uses, and the participant’s right to decline any question or withdraw at any time without penalty or loss of entitlements. To protect privacy, interviews were conducted in a private area of the participant’s home and in their preferred language (Sarawakian Malay dialect, Iban, or Mandarin). All study materials used unique study IDs only; names and direct identifiers were stored separately from survey data. Completed forms were kept physically secure in the field and transferred daily to an encrypted server with role-based access control. We applied de-identification at source, implemented range/logic checks to prevent accidental disclosure, and restricted access to only the personnel required for monitoring and analysis. Field staff were trained in neutral prompting, confidentiality, and participant safety (including procedures for pausing/terminating interviews if distress was observed). The protocol prespecified that no coercive incentives would be used and that any complaints or adverse events would be documented and reported to the investigators and ethics committee.

### Reporting guideline

This manuscript adheres to the STROBE (Strengthening the Reporting of Observational Studies in Epidemiology) recommendations for cross-sectional studies: we pre-specified the analysis plan, described the design, setting, eligibility and recruitment, clearly defined exposures, outcomes, confounders, and effect modifiers, detailed bias mitigation, sample-size rationale, statistical methods (including handling of missing data and model diagnostics), and presented effect estimates with 95% CIs alongside a participant-flow description; a completed STROBE checklist is provided in the Supplementary material and items were cross-checked against the EQUATOR Network guidance^28,29^.

## Results

### Participant characteristics and bivariate associations with protective self-care behaviours during haze

We analyzed 481 respondents drawn from the state-wide. The age distribution comprised 22.5% of younger adults (18–29 years), 53.2% of adults (30–45 years), and 24.3% of older adults (> 40 years), with a mean (SD) age of 39.5 (12.0) years (Table 1). Most participants were male (64.7%), had at least secondary education (79.4%), and reported formal employment (59.5%). The median (IQR) monthly household income was MYR 1,700 (1,325–2,800), and approximately one in four households (with 7 or more members) met the criterion for a large household. Air-purifier ownership was uncommon, with 78.0% reporting no purifier at home. Smoking prevalence was 35.8%. Regarding morbidity, 70.5% reported no diagnosed medical illness, 12.9% reported one, and 16.6% reported two or more conditions. Regarding service accessibility, 45.1% of respondents lived within 10 km of the nearest health facility, while the remainder were farther away, indicating heterogeneous access across settings.

**Table 1:** Sociodemographic characteristics of the respondents (n = 481).

Sociodemographic characteristics	*n (%)*	Protective Self-care behaviour			χ^2^(df)	*P* value
		Poor	Moderate	Good		
Age (years old)					40.01(4)	< 0.001
18–29 (Younger adults)	108(22.5)	35(32.4)	29(26.9)	44(40.7)		
30–45 (Adults)	256(53.2)	69(27.0)	129(50.3)	58(22.7)		
> 40 (Older age)	117(24.3)	17(14.6)	79(67.5)	21(17.9)		
Mean (SD) Median (IQR)	39.5 (11.96) 39.0 (30–45)					
Gender					5.94(2)	0.051
Male	311(64.7)	68(21.9)	164(52.7)	79(25.4)		
Female	170(35.3)	53(31.2)	73(42.9)	44(25.9)		
Education level					28.12(2)	< 0.001
Primary and lower	99(20.6)	16(16.2)	72(72.7)	11(11.1)		
Secondary and above	382(79.4)	105(27.5)	165(43.2)	112(29.3)		
Occupation					25.27(4)	< 0.001
Not in the labour force	81(16.8)	26(32.1)	40(49.4)	15(18.5)		
Self-sustained job	114(23.7)	18(15.8)	77(67.5)	19(16.7)		
Formal employment	286(59.5)	77(26.9)	120(49.3)	89(25.6)		
Marital status					41.40(2)	< 0.001
Married	355(73.8)	52(41.3)	32(25.4)	42(33.3)		
Unmarried	126(26.2)	69(19.4)	205(57.7)	81(22.8)		
Household size					33.33(4)	< 0.001
Small (< 5 members)	174(36.2)	29(16.7)	109(62.6)	36(20.7)		
Medium (5–6 members)	187(38.9)	46(24.6)	93(49.7)	48(25.7)		
Large (≥ 7 members)	120(24.9)	46(38.3)	35(29.2)	39(32.5)		
Household income level (MYR)					57.01(6)	< 0.001
RM1000 and below	91(18.9)	11(12.1)	68(74.7)	12(13.2)		
1001–2000	216(44.9)	45(20.8)	118(54.6)	53(24.5)		
2001–3000	89(18.5)	37(41.6)	25(28.1)	27(30.3)		
More than RM3000	85(17.7)	28(32.9)	26(30.6)	31(36.5)		
Mean (SD) Median (IQR)	2154.4 (1804.49) 1700(1325 – 2800)					
Medical problems					9.49(2)	0.009
No	331(43.2)	79(23.3)	160(47.2)	100(29.5)		
Yes	435(56.8)	42(29.6)	77(54.2)	23(16.2)		
Distance to nearest health facilities					56.24(2)	< 0.001
10 km and less	217(45.1)	24(11.1)	110(50.7)	83(38.2)		
More than 10 km	264(54.9)	97(36.7)	127(48.1)	40(15.2)		
Air purifier at home					26.61(2)	< 0.001
Yes	106(22.0)	43(40.6)	30(28.3)	33(31.1)		
No	375(78.0)	78(20.8)	207(55.2)	90(24.0)		
Smoker					4.62(2)	0.099
Yes	172(35.8)	37(21.5)	96(55.8)	39(22.7)		
No	309(64.2)	84(27.2)	141(45.6)	84(27.2)		
Number of medical illnesses					9.52(4)	0.049
No illness	339(70.5)	79(23.3)	160(47.2)	100(29.5)		
1 medical illness	62(12.9)	18(29.0)	34(54.8)	10(16.1)		
2 or more	80(16.6)	24(30.0)	43(53.8)	13(16.3)		

*Descriptive statistics with chi-square tests for associations with self-care behaviour category; *p* < 0.05 considered statistically significant. Percentages under Poor/Moderate/Good are row-based (within each subgroup). χ2 = chi-square; df = degrees of freedom.

In bivariate analyses (chi square tests), self-care classification (Poor/Moderate/Good) varied significantly by age (*p* < 0.001), occupation (*p* < 0.001), marital status (*p* < 0.001), household size (*p* < 0.001), household income (*p* < 0.001), home air-purifier ownership (*p* < 0.001), and distance to the nearest health facility (*p* < 0.001). Overall, respondents with higher incomes, formal employment, and purifier ownership were more frequently represented in the ‘Good self-care’ category, consistent with greater resource availability for protective actions (for instance, acquiring respirators, limiting exposure indoors). Larger households exhibited a more dispersed distribution across categories, suggesting competing demands or mixed capacities within multi-person households. In contrast, those closer to health facilities showed a shift toward better self-care, plausibly reflecting easier access to guidance and supplies. Medical illness demonstrated a non-significant trend (*p* = 0.090), with no clear separation across morbidity strata, and smoking was also non-significant (*p* = 0.099). Taken together, these patterns suggest that socio-economic position and service accessibility are significant correlates of protective self-care behaviours during haze. In contrast, current smoking status and comorbidity burden do not differentiate self-care categories at conventional thresholds in this dataset.

### Profiles of determinants: haze tolerance, political trust, perceived costs, and knowledge

Across domains, respondents displayed cautious behavioural norms alongside high institutional confidence (Table 2). Although nearly half expressed general acceptance of “haze weather,” they overwhelmingly rejected unprotected outdoor activity: most disagreed with going outside without a mask and with exercising outdoors during haze, and acceptance of having no indoor air purifier was limited, together indicating normative caution despite ambivalence toward haze as a condition. In contrast, political trust was broadly favourable: large majorities agreed the government has the capacity to control haze, that official pollution data are transparent, and that current measures are appropriate, with similarly high expectations for future innovation.

**Table 2:** Haze tolerance, protective measures, political trust, cost perception, and knowledge of haze.

Domains/Items	Strongly Disagree n (%)	Disagree n (%)	Neutral n (%)	Agree n (%)	Strongly Agree n (%)
Haze tolerance (Weather and Protective measures)					
I think haze weather all this time is acceptable	3(0.6)	29(6.0)	213(44.3)	224(46.6)	12(2.5)
I can accept going out without a mask on haze days	78(16.2)	234(48.6)	73(15.2)	85(17.7)	11(2.3)
I can accept outdoor exercise on haze days	142(29.5)	228(47.4)	49(10.2)	52(10.8)	10(2.1)
I can accept that there is no indoor air purifier on hazy days	42(8.7)	168(34.9)	140(29.1)	120(24.9)	11(2.3)
Political trust (control capacity, pollution data, and control measures)					
I think the government has the capacity to control haze	2(0.4)	12(2.5)	71(14.8)	217(45.1)	179(37.2)
I think the official haze pollution data are open and transparent	0(0.0)	10(2.1)	99(20.6)	181(37.6)	191(39.7)
I think the current haze control policies and measures are appropriate and trustworthy	0(0.0)	10(2.1)	95(19.8)	208(43.2)	168(34.9)
I think the government will facilitate the innovation of haze control policies and measures in the future	0(0.0)	4(0.8)	119(24.7)	180(37.4)	178(37.0)
Cost perception (economic cost and traffic cost)					
Haze control will reduce employment opportunities	9(1.9)	31(6.4)	177(36.8)	208(43.2)	56(11.6)
Haze control is not conducive to local economic development	10(2.1)	44(9.1)	188(39.1)	176(36.6)	63(13.1)
Haze control will bring traffic inconvenience to people’s travel	2(0.4)	50(10.4)	154(32.0)	216(44.9)	59(12.3)
Knowledge of Haze (acquisition intention and knowledge level)					
I want to get haze pollution data every day	1(0.2)	16(3.3)	28(5.8)	247(51.4)	189(39.3)
I want to know the causes of haze and its impact on human health	0(0.0)	14(2.9)	29(6.0)	235(48.9)	203(42.2)
I want to know the current control strategy for haze pollution	0(0.0)	9(1.9)	27(5.6)	248(51.6)	197(41.0)
I often learn about haze information by surfing the Internet, watching TV, or reading newspapers	3(0.6)	11(2.3)	79(16.4)	193(40.1)	195(40.5)
I have substantial knowledge of haze (source, formation mechanism, and impacts)	7(1.5)	28(5.8)	208(43.2)	94(19.5)	144(29.9)

At the same time, cost salience was notable and may temper protective action and policy acceptance: most respondents agreed that control efforts could reduce employment opportunities and disrupt mobility/traffic, and about half viewed control as unfavourable for local economic development, with substantial neutrality rather than outright disagreement. Information dynamics exhibited a distinctive profile, characterized by a very high intention to acquire information (daily data, causes/impacts, and control strategies) and frequent media use, coupled with moderate self-rated knowledge, suggesting a motivation and confidence gap that risk communication can target. Overall, the pattern is characterized by low tolerance for risk-taking behaviours, high trust, salient perceived costs, and strong information-seeking, an actionable combination for tailoring messaging and support.

### Core community resilience profile

Across all four subdomains, connection and caring, resources, transformative potential, and disaster management, responses clustered toward agreement, indicating a generally resilient community context (Table 3). Indicators of social cohesion were robust: majorities agreed that neighbours *feel they belong* (58.8%) and are *committed to neighbourhood well-being* (67.6%), with similarly high agreement that *people help each other* (67.4%) and that *fair treatment regardless of background* is the norm (52.4%; 11.0% strongly agree). Perceptions of governance and access were also favourable: most respondents agreed that the neighbourhood *has effective leaders* (54.9%), *knows where to get things done* (53.2%), and *can obtain needed services* (53.8%). Notably, however, neutral selections remained sizeable for several resource items (≈34–38%), suggesting uneven visibility or reliability of tangible supports even where leadership and service navigation are viewed positively.

**Table 3:** Core community resilience.

Domains/Items	Strongly Disagree n (%)	Disagree n (%)	Neutral n (%)	Agree n (%)	Strongly Agree n (%)
Connection and caring					
People in my neighborhood feel like they belong to the neighbourhood	2(0.4)	17(3.5)	138(28.7)	283(58.8)	41(8.5)
People in my neighborhood are committed to the well-being of the neighborhood	0(0.0)	12(2.5)	102(21.2)	325(67.6)	42(8.7)
People in my neighborhood have hope about the future	1(0.2)	9(1.9)	174(36.2)	258(53.6)	39(8.1)
People in my neighborhood help each other	1(0.2)	14(2.9)	101(21.0)	324(67.4)	41(8.5)
My neighborhood treats people fairly no matter what their background is	0(0.0)	12(2.5)	164(34.1)	252(52.4)	53(11.0)
Resources					
My neighborhood has the resources it needs to take care of neighborhood problems	9(1.9)	21(4.4)	183(38.0)	245(50.9)	23(4.8)
My neighborhood has effective leaders	1(0.2)	29(6.0)	165(34.3)	264(54.9)	22(4.6)
People in my neighborhood are able to get the services they need	1(0.2)	22(4.6)	174(36.2)	259(53.8)	25(5.2)
People in my neighborhood know where to go to get things done	8(1.7)	8(1.7)	186(38.7)	256(53.2)	23(4.8)
Transformative potential					
My neighborhood works with organizations and agencies outside the neighborhood to get things done	5(1.0)	11(2.3)	105(21.8)	284(59.0)	76(15.8)
People in my neighborhood communicate with leaders who can help improve the neighborhood	1(0.2)	14(2.9)	128(26.6)	272(56.5)	66(13.7)
People in my neighborhood are aware of neighborhood issues that they might address together	4(0.8)	14(2.9)	118(24.5)	295(61.3)	50(10.4)
People in my neighborhood discuss issues so they can improve the neighborhood	2(0.4)	6(1.2)	125(26.0)	293(60.9)	55(11.4)
People in my neighborhood work together on solutions so that the neighborhood can improve	0(0.0)	10(2.1)	120(24.9)	289(60.1)	62(12.9)
Disaster management					
My neighborhood looks at its successes and failures so it can learn from the past	1(0.2)	13(2.7)	116(24.1)	318(66.1)	33(6.9)
My neighborhood develops skills and finds resources to solve its problems and reach it goals	2(0.4)	25(5.2)	120(24.9)	304(63.2)	30(6.2)
My neighborhood has priorities and sets goals for the future	0(0.0)	14(2.9)	172(35.8)	277(57.6)	18(3.7)

Signals of collective action capacity were consistently high. Clear majorities reported that residents *work with outside organisations to get things done* (59.0%, with 15.8% strongly agreeing), communicate with leaders to improve the neighbourhood (56.5%), discuss issues (60.9%), and *work together on solutions* (60.1%). In the disaster management domain, most agreed that the neighbourhood *learns from past successes and failures* (66.1%) and *develops skills/resources to solve problems* (63.2%), with more than half endorsing the presence of *future priorities and goal setting* (57.6%). Taken together, the pattern depicts a high-trust, action-oriented social environment with strong cohesion and problem-solving norms; the principal gap lies in material resources, where substantial neutrality points to scope for strengthening the visibility and reach of concrete supports that enable communities to translate intent into sustained preparedness and protective action.

### Determinants and protective self-care behaviours during haze across Sarawak’s three zones: Southern, Central, and Northern

Determinant profiles and the primary outcome varied significantly by zone (Table 4). Mean haze tolerance was highest in the Southern zone and lowest in the Northern zone (10.93 vs 10.05; *p* = 0.036). Political trust and knowledge showed pronounced Southern advantages (trust: 17.55 vs. 14.54–14.68; knowledge: 21.94 vs. 18.06–19.12; both *p* < 0.001). Perceived costs varied in the opposite direction, being lowest in the South (6.41) and higher in the Central/Northern regions (9.1; *p* < 0.001). Community resilience was graded Southern → Central → Northern (64.71, 61.91, 58.57; *p* < 0.001). Despite these patterns, protective self-care behaviours during haze was highest in the Northern region (88.79) and lowest in the Central region (83.83), with the Southern region intermediate (86.62; *p* < 0.001).

**Table 4:** Protective self-care behaviours during haze and other determinants according to zones in Sarawak.

Domain	Overall	Southern	Central	Northern	*p* value
Haze tolerance	10.71(2.80)	10.93(3.00)	10.51(2.22)	10.05(2.48)	0.036
Political trust	16.53(2.86)	17.55(2.77)	14.54(1.94)	14.68(1.80)	< .001
Cost perception	7.36(2.29)	6.41(1.83)	9.11(2.22)	9.15(1.69)	< .001
Knowledge	20.76(3.50)	21.94(3.39)	18.06(2.76)	19.12(1.91)	< .001
Community resilience	63.20(7.86)	64.71(7.95)	61.91(7.44)	58.57(5.71)	< .001
Protective self-care behaviours during haze	86.45(8.64)	86.62(9.47)	83.83(6.59)	88.79(6.11)	< .001

*Values are presented as mean (SD). *p-*values were obtained using one-way ANOVA for comparisons across regions.

Taken together, the Southern zone concentrates higher trust, knowledge, community resilience, and haze tolerance alongside lower perceived costs, yet the Northern zone achieves the highest self-care scores, suggesting that contextual or experiential factors (recent exposure history, local norms, logistical constraints) may enable effective protective action even where institutional confidence and knowledge scores are lower. Conversely, the Central zone combines higher perceived costs with lower trust/knowledge and the lowest self-care, indicating a potential target for intensified risk communication and practical support to reduce barriers to protective behaviour. These zonal contrasts motivate formal multivariable analyses and interaction tests to evaluate whether perceived costs, trust, knowledge, or community resilience modify the relationship between exposure contexts and self-care. To assess potential confounding by zone, we refitted the multivariable model with zone included as an additional covariate. The key associations were modestly attenuated, indicating that the main conclusions were not driven solely by regional composition.

### Ordinal associations with protective self-care behaviours during haze category (proportional-odds models)

In the sociodemographic model, as indicated in Table 5, proximity to a health facility emerged as the strongest correlate of higher self-care category: respondents living ≤ 10 km from a facility had 3.38-fold higher odds of being in a better self-care category than those > 10 km (β = 1.217; OR = 3.378; 95% CI: 2.165–5.272; *p* < 0.001). Being unmarried was associated with lower odds of higher self-care (β =  − 0.740; OR = 0.477; 95% CI: 0.279–0.817; *p* = 0.007). Other covariates, including age group, gender, education, occupation, income, household size, air purifier ownership, smoking, and self-reported medical illness, were not statistically significant. These results suggest that service accessibility and household social status (marital status) differentiate self-care categorisation, while measured socio-economic and morbidity factors do not show independent associations in this specification. In the adjusted ordinal model, an odds ratio below 1 indicates lower cumulative odds of being in a higher self-care category (relatively greater likelihood of being in lower categories), after accounting for the covariates included in the model. Therefore, the adjusted association for unmarried status should be interpreted as an association with a lower self-care category ranking, even if the unadjusted/descriptive distribution appears different.

**Table 5:** Sociodemographic and access correlates of self-care category, proportional-odds ordinal logistic regression.

Variable	Level	β	OR	95% CI		*p value*
				Lower	Upper	
Age	Young	0.296	1.345	0.629	*2.873*	0.445
	Adults	− 0.283	0.753	0.419	*1.355*	0.344
	Older age group	Ref				
Gender	Male	0.177	1.193	0.734	1.940	0.476
	Female	Ref				
Marital status	Unmarried	− 0.740	0.477	0.279	0.817	0.007
	Married	Ref				
Education	Primary & lower	− 0.356	0.700	0.377	1.302	0.261
	Secondary & above	Ref				
Occupation	Not employed	− 0.362	0.696	0.368	1.315	0.264
	Self-sustained	0.312	1.367	0.759	2.460	0.298
	Formal employment	Ref				
Income group	≤ RM1000	− 0.057	0.944	0.419	2.125	0.890
	RM1001-2000	− 0.015	0.985	0.542	1.791	0.960
	RM2001-3000	− 0.318	0.727	0.381	1.389	0.335
	> RM3000	Ref				
Household size	≤ 4	0.164	1.178	0.695	1.996	0.542
	5–6	.108	1.114	0.675	1.840	0.672
	≥ 7	Ref				
Health facility distant	≤ 10 km	1.217	3.378	2.165	5.272	0.000
	> 10 km	Ref				
Air purifier	Yes	− 0.382	0.683	0.422	1.105	0.120
	No	Ref				
Smoking	Yes	0.122	1.130	0.714	1.788	0.601
	No					
Medical illness	No	− 0.319	0.727	0.370	1.429	0.355
	Yes	Ref				

Odds ratios represent cumulative odds of being in a higher self-care category (Poor → Moderate → Good).

In the determinants model with continuous domain scores (Table 6), higher haze tolerance (per-unit increase) was associated with higher self-care category (β = 0.166; OR = 1.181; 95% CI: 1.094–1.275; *p* < 0.001), as was higher knowledge (β = 0.164; OR = 1.178; 95% CI: 1.070–1.297; *p* = 0.001). Community resilience showed a small inverse association (β =  − 0.040; OR = 0.961; 95% CI: 0.931–0.992; *p* = 0.013), whereas political trust (β =  − 0.100; OR = 0.905; 95% CI: 0.808–1.014; *p* = 0.085) and perceived costs (β = 0.067; OR = 1.069; 95% CI: 0.958–1.193; *p* = 0.231) were not significant. Taken together, the ordinal-logit results indicate that information and attitudinal readiness (knowledge and tolerance) are positively associated with a more favourable self-care classification, independent of covariates, while the modest inverse association with community resilience warrants further exploration.

**Table 6:** Haze-related determinants and community resilience associated with protective self-care behaviours during haze category, proportional-odds ordinal logistic regression adjusted for sociodemographics.

Variable	β	AOR	95% CI		*p value*
			Lower	Upper	
Haze tolerance	0.166	1.181	1.094	1.275	0.000
Political trust	− 0.100	0.905	0.808	1.014	0.085
Cost perception	0.067	1.069	0.958	1.193	0.231
Knowledge	0.164	1.178	1.070	1.297	0.001
Community resilience	− 0.040	0.961	0.931	0.992	0.013

Odds ratios from proportional-odds ordinal logistic regression represent cumulative odds of being in a higher self-care category (Poor → Moderate → Good). Estimates in Table 6 are adjusted odds ratios (AORs) controlling for sociodemographic and access covariates as specified in the model.

The Test of Parallel Lines indicated violation of the proportional-odds assumption (LR χ^2^(5) = 88.33, *p* < 0.001). Therefore, multinomial (generalised logit) regression was conducted as a sensitivity analysis to estimate threshold-specific associations; results are provided in Supplementary Table S1. The multinomial model showed that several predictors differed in strength across thresholds, supporting cautious interpretation of proportional odds estimates as summary associations.

## Discussion

In our state-representative sample, the prevalence of “Good” self-care during haze was 25.6%. In comparison, *Moderate* accounted for 49.3%, implying that roughly three in four adults are not yet in the highest, most protective band. This distribution suggests that many residents may be attempting some recommended actions but are not consistently achieving the full set of protective behaviours needed to reach the “Good” category. In a comparative context, this profile is concerning but consistent with regional literature, which shows that robust protective self-care behaviours during hazes (for example, consistent N95 use, sustained indoor air quality control) rarely exceed one-third to one-half of adults, even under severe haze, with uptake patterns influenced by risk perception, knowledge, and perceived efficacy^30,31^. Moreover, public health guidance emphasizes that the proper fit and use of respirators are critical for effective particulate filtration. This implementation hurdle likely constrains the share that reaches *good* practice in real-world conditions^32^. Taken together, the dominance of *Moderate* and relatively small, good stratum signal substantial headroom for improvement via targeted risk communication and practical enablers (affordable, well-fitting respirators; access to clean-air options), particularly for groups facing informational or material barriers.

The zonal contrasts and ordinal associations align with a growing body of work demonstrating that risk perception, knowledge, and practical access jointly influence the adoption of air-pollution protective behaviours. In our data, higher knowledge and the tolerance construct (as operationalised in this study) were associated with better self-care classification, mirroring synthesis evidence that risk appraisal and perceived efficacy are consistent antecedents of protective action in air-pollution contexts^33^. The interpretation of haze tolerance and community resilience should be approached with caution because both are scale-based constructs, operationalised in this study, and may partly reflect overlapping psychosocial contexts (perceived coping, acceptance, and feasibility) rather than discrete causal pathways. Accordingly, observed associations should be interpreted as correlates that may help prioritise further investigation and intervention design, rather than as direct effects. This finding reinforces the idea that “know-what” (general awareness) is insufficient without “know-how” (behaviourally specific steps, such as correct respirator fit-checking and feasible indoor air strategies). At the same time, political trust, high at the descriptive level, did not independently predict higher self-care in the adjusted ordinal model, suggesting either context-dependency or mediation through message uptake rather than direct behavioural effects; this nuance is compatible with recent experimental and observational studies in public-health policy that find trust enables compliance when paired with clear, actionable guidance and credible messengers^34,35^. In Malaysia, policy analyses continue to highlight communication and access gaps during haze alerts, suggesting that even with positive institutional sentiment, material enablers (for example, affordable respirators, clean-air options, service reach) remain crucial for converting intent into practice^36^.

Two additional patterns merit clarification. First, the strong association between proximity to health facilities and higher self-care categories is consistent with Malaysian evidence that haze episodes increase healthcare utilization, particularly for respiratory morbidity, thereby making facility touchpoints important channels for timely risk communication and supply distribution (such as respirators) ^37^. In practical terms, this also implies that districts with poorer geographic access may require alternative delivery channels (mobile outreach, community-based distribution, and pre-positioned supplies) to avoid widening protective inequities during haze surges. Second, we observed a small inverse association between community resilience scores and the self-care category after adjusting for confounders. This association is unlikely to be due to collinearity with related psychosocial constructs, as variance inflation factors were below conventional thresholds. Therefore, we interpret this inverse association cautiously as potentially reflecting construct-level differences (community-level coping perceptions versus individual protective routines), contextual access constraints, or behavioural substitution mechanisms rather than a statistical artefact. While counterintuitive, this is not inconsistent with resilience scholarship: composite resilience captures collective capacities (coordination, leadership, resource navigation) that may substitute for individual self-protection in some settings (reliance on community shelters, shared air-cleaning spaces), and domain weighting, measurement timing, or ceiling effects can yield attenuated or direction-reversing associations with individual behaviours^38,39^. This warrants follow-up using domain-specific resilience models and mediation tests (resilience → trust/knowledge → behaviour) rather than a single global score. Notably, the inverse community resilience association was most apparent for *Good vs Poor* in multinomial sensitivity analyses, suggesting this relationship may operate primarily at the transition to the highest self-care level rather than uniformly across all thresholds.

Our prevalence estimates, which indicate only one-quarter in the good self-care band, also need to be interpreted considering cost and implementability constraints. Even when knowledge is high, equipment and comfort barriers limit sustained respirator use; economic evaluations indicate that achieving meaningful filtration requires consistent, well-fitted N95s, with non-trivial costs when scaled to households and repeated episodes^40^. Complementary communication research emphasizes that behaviourally informed messaging, delivered at exposure thresholds and paired with tangible enablers, outperforms generic advisories. This approach could be operationalized in Sarawak using zonal API triggers and targeted distribution to high-burden, low-resource districts^35,41^. Accordingly, programme designs that treat respirator access and “clean-air options” as routine preparedness inputs, rather than ad hoc emergency supplies, are more likely to shift residents from Moderate to Good practice.

Zonal differences in self-care likely reflect contextual heterogeneity in exposure experience and feasibility constraints rather than demographics alone. The Northern zone’s comparatively higher self-care, despite lower trust/knowledge scores, may reflect experience-driven adaptation, consistent with the preparedness literature, which shows that repeated hazard salience can catalyse protective routines when cues are timely and actionable^42^. Consistent with this interpretation, our findings support using shared health-protective triggers while tailoring delivery channels and enablement strategies to local feasibility in urban versus rural/remote settings. In interpreting inter-annual patterns, it is also vital to note Malaysia’s 2018 transition to a PM2.5-inclusive API, which improved alignment with contemporary health evidence and strengthens the case for focusing on short-term PM2.5-related thresholds in behavioural messaging^2,17,43^. These shifts align with the WHO 2021 Air Quality Guidelines, which emphasize substantially lower health-protective concentrations for PM2.5 than legacy standards, underscoring that the *Moderate* and *Good* practice bands we observed are not merely desirable but necessary to reduce exposure during haze in a meaningful way^44,45^. Together, the evidence supports targeting zone-specific triggers and pairing them with access enablers (respirators, clean-air options) to convert intention into action where trust or knowledge alone is insufficient^42,46^. Given Sarawak’s highly heterogeneous settlement patterns, the most effective “trigger-message-enabler” bundle will likely require local tailoring (for example, urban vs. rural/remote feasibility), while preserving consistent health-protective thresholds and core behavioural recommendations.

A key strength is the state-representative, multilingual household survey spanning Sarawak’s three zones, coupled with domain-based measurement of tolerance, trust, perceived costs, knowledge, community resilience, and Likert-anchored outcome scoring for self-care. Methodologically, we prespecified design-aware estimation, evaluated internal consistency/structure, and conducted robustness checks to enhance interpretability. A further strength of our approach is modelling the primary outcome as ordinal (Poor/Moderate/Good) rather than forcing a binary cut. Ordinal models preserve the rank information inherent in Likert-anchored categories and typically offer greater statistical efficiency and power than dichotomisation, which discards within-scale information^47^. They also provide a single, easily interpretable cumulative odds ratio that summarizes a movement toward more protective categories while allowing extensions when assumptions may not be fully satisfied. Since the proportional-odds model estimates cumulative odds, these associations should be understood as relative shifts toward higher self-care categories, rather than as absolute changes or causal effects. Accordingly, the practice implications were presented as implementation hypotheses informed by observed associations and should be evaluated in future studies. In our dataset, the Test of Parallel Lines indicated that the proportional-odds assumption was not fully met, implying that some predictors may vary in strength across thresholds. We therefore interpret proportional-odds estimates as summary associations and present multinomial (generalised logit) sensitivity results to provide threshold-specific estimates. Notably, the direction and significance of key predictors were consistent across sensitivity analyses (multinomial models and analyses using the continuous self-care score), strengthening confidence that the main inferences are not driven solely by outcome categorisation or a single modelling specification. Nevertheless, given the cross-sectional design, all findings should be interpreted as associations rather than causal effects, and implementation recommendations should be evaluated prospectively.

Nevertheless, this study was cross-sectional and cannot establish causality. Self-report introduces potential desirability and recall biases, and API is a composite indicator with limited pollutant specificity (though its post-2018 PM2.5 basis improves relevance), which limits direct comparability of longer-term API trends across pre- and post-2018 periods. Our quartile categorisation aids communication but inevitably sacrifices some granularity relative to continuous scores. Quartile-based categorisation is sample-dependent and may limit comparability across studies^48^. However, these quartile-based categories remain useful for practical planning because they identify relative “lower practice” strata within the surveyed population that can be prioritised for targeted enablement (respirator access, fit guidance, and feasible clean-air options) and behaviourally specific ‘how-to’ messaging. These groupings should not be interpreted as guideline-defined thresholds or as absolute prevalence estimates that can be compared directly across settings. Finally, although we addressed missing data using principled approaches, residual confounding and unmeasured determinants, such as building characteristics and employer policies, remain possible^49^. We also note that while the survey was designed to be state-representative, future analyses could further explore whether self-care patterns differ across Sarawak’s major ethnic and livelihood groups to strengthen equity-targeted implementation planning. Apparent discrepancies between descriptive proportions and adjusted associations can arise because adjustment accounts for covariate imbalance (age structure, access to facilities, and other determinants), which may shift the direction or magnitude of associations compared with unadjusted comparisons.

Based on our findings, recommendations were refined to target the key modifiable and feasibility-related determinants of protective self-care. Specifically, because higher knowledge was associated with higher self-care, risk communication should move beyond awareness messages and deliver behaviourally specific “how-to” content (for example, correct respirator type selection and fit-checking, practical indoor air strategies during peak API). Because access constraints and facility proximity were associated with self-care, preparedness should include pre-positioned supplies (respirators) and delivery through primary care facilities, mobile outreach, and community-based distribution in low-access districts. Finally, given the heterogeneity across Sarawak’s zones, implementation should use the same health-protective triggers but tailor delivery channels and enablement strategies to urban versus rural/remote feasibility. Translating these findings into practice suggests three priorities. First, adopt exposure-triggered communication keyed to recent API thresholds, delivered through trusted local channels and coupled with “how-to” guidance that emphasises fit-correct respirator use and practical indoor air improvements^50^. Second, pair messages with material enablers, such as subsidized or pre-positioned N95s and portable filtration, directed to low-resource hotspots, reflecting the equity gaps highlighted in smoke/haze risk-communication reviews^42,51^. Based on the observed role of knowledge in predicting higher self-care, interventions should prioritise behaviourally specific risk communication (“how-to” components) rather than awareness-only messages, for example, correct respirator type selection, fit-checking, safe activity modification during peak API, and feasible indoor air strategies. Third, treat health-facility proximity as an operational asset: integrate point-of-care prompts and supplies (mask fit cards, brief counselling) during haze-responsive service surges, leveraging facilities as distribution and feedback nodes for community-tailored messaging. The threshold-specific patterns observed in sensitivity analyses also suggest that strategies to shift residents from Poor to Moderate (addressing trust and affordability barriers) may differ from those needed to move from Moderate to Good (actionable know-how and practical capability), reinforcing the value of bundled message-enabler approaches.

The inverse association between the community resilience score and the higher self-care category was counterintuitive and should be interpreted cautiously. This finding is exploratory and may reflect construct-level differences (perceived collective coping capacity versus individual protective routines), contextual feasibility constraints, or behavioural substitution (for example, reliance on communal coping strategies rather than individual actions), rather than a true negative effect of resilience. The inverse association also indicates that public health planning should not assume that perceived collective coping automatically translates into individual protective routines; programmes should therefore include explicit individual-level prompts and enablement alongside community-level mobilisation. As political trust did not independently predict self-care after adjustment, strategies may need to emphasise practical feasibility, clear triggers, consistent messaging, and access to protective resources, rather than relying on institutional trust alone to drive behaviour change.

As regional air-quality standards converge with WHO guidance, aligning triggers, messages, and enablers may help shift the proportion of residents achieving “Good” practice during seasonal haze^44,50,51^. Feasibility will vary across Sarawak, particularly in rural and remote settings where travel time to clinics is substantial. In these contexts, the most immediately actionable element is triggered risk communication using existing district and community networks (Health Clinics outreach, community leaders, schools, and WhatsApp services/radio channels), paired with concise ‘how-to’ guidance (fit-checks, indoor air improvement steps). Respirator provision can be operationalised through district-level pre-positioning and distribution via primary care facilities and mobile outreach during haze surges, prioritising low-resource localities. Where household filtration is not feasible, interim clean-air spaces (designated rooms in clinics, community halls, or schools with basic filtration) may be a pragmatic option during peak episodes. To maximise value-for-money, implementation should prioritise the highest-yield combination of (i) trigger-based messaging, (ii) respirator access with fit guidance, and (iii) feasible clean-air options, with monitoring indicators mapped to shifts across Poor → Moderate → Good bands. These recommendations are intended as an implementation-ready starting point and should be refined through operational pilots and evaluation.

## Conclusions

In this state-representative survey, protective self-care during seasonal haze was patterned by modifiable psychosocial determinants and feasibility constraints. Strengthening exposure-triggered risk communication with behaviourally specific ‘how-to’ guidance, alongside practical enablement (respirator access, fit guidance, and feasible clean-air options), may help shift more residents toward higher protective practice, particularly in low-access settings. Future longitudinal and implementation studies are warranted to assess causality and evaluate targeted strategies across Sarawak’s zones.

## Supplementary Information

Below is the link to the electronic supplementary material.

Supplementary Material 1

## Data Availability

All data generated or analysed during this study are included in this published article.
