# Supplementary material for: Haze-protective self-care behaviours in Sarawak, Malaysia: a state-representative cross-sectional study
Source: Sci Rep. 2026 Apr 20;16:18384. doi: 10.1038/s41598-026-49870-9 (PMC13265957; doi:10.1038/s41598-026-49870-9)
Supplement: Supplementary file 1 — Supplementary Material 1 [file 41598_2026_49870_MOESM1_ESM.docx]

**Multinomial logistic regression sensitivity analysis of the self-care behavior category**

Given violation of the proportional-odds assumption in the ordinal model (Test of Parallel Lines: LR χ² (5) = 88.33, p<0.001), we conducted multinomial (generalised logit) regression as a sensitivity analysis to estimate threshold-specific associations. Relative to the Poor category, the odds of being in the Moderate category were higher with greater cost perception (OR=1.337, 95% CI 1.167–1.532; p<0.001) and lower with higher political trust (OR=0.814, 95% CI 0.715–0.927; p=0.002), whereas tolerance, haze knowledge, and resilience were not statistically significant for this contrast. In contrast, the odds of being in the Good category (vs Poor) were higher with higher tolerance score (OR=1.271, 95% CI 1.146–1.410; p<0.001), higher cost perception (OR=1.293, 95% CI 1.108–1.509; p=0.001), and greater haze knowledge (OR=1.324, 95% CI 1.147–1.527; p<0.001), while community resilience showed a small inverse association (OR=0.943, 95% CI 0.902–0.985; p=0.008). Overall, key predictors of achieving the highest self-care category remained evident, while several effects varied across thresholds, supporting presentation of multinomial results as a robustness check and interpretation of proportional odds estimates as summary associations.

**Supplementary Table S1. Multinomial logistic regression sensitivity analysis of self-care behavior category (reference category = Poor).**

| **Predictor** | **Moderate vs Poor OR (95% CI)** | **p-value** | **Good vs Poor OR (95% CI)** | **p-value** |
| --- | --- | --- | --- | --- |
| Haze tolerance score | 0.956 (0.861–1.061) | 0.398 | 1.271 (1.146–1.410) | <0.001 |
| Political trust total score | 0.814 (0.715–0.927) | 0.002 | 0.878 (0.747–1.030) | 0.111 |
| Cost perception score | 1.337 (1.167–1.532) | <0.001 | 1.293 (1.108–1.509) | 0.001 |
| Haze knowledge score | 1.067 (0.953–1.193) | 0.260 | 1.324 (1.147–1.527) | <0.001 |
| Community resilience total score | 0.980 (0.943–1.019) | 0.310 | 0.943 (0.902–0.985) | 0.008 |

**Notes:** Reference outcome category = Poor. Model fit: likelihood ratio χ² (10) = 151.56, p<0.001. Pseudo-R²: Cox & Snell=0.270; Nagelkerke=0.308; McFadden=0.151.

Outcome categories: Moderate vs Poor and Good vs Poor. Estimates are odds ratios (OR) with 95% confidence intervals.
